# Performance of the marginal structural cox model for estimating individual and joined effects of treatments given in combination

**DOI:** 10.1186/s12874-017-0434-1

**Published:** 2017-12-04

**Authors:** Clovis Lusivika-Nzinga, Hana Selinger-Leneman, Sophie Grabar, Dominique Costagliola, Fabrice Carrat

**Affiliations:** 1 Sorbonne Universités, INSERM, UPMC Université Paris 06, Institut Pierre Louis d’épidémiologie et de Santé Publique (IPLESP UMRS 1136), Paris, France; 2 Unité de Biostatistique et d’épidémiologie Groupe hospitalier Cochin Broca Hôtel-Dieu, Assistance Publique Hôpitaux de Paris (AP-HP), and Université Paris Descartes, Sorbonne Paris Cité, Paris, France; 3 Unité de Santé Publique, Hôpital Saint-Antoine, Assistance Publique-Hôpitaux de Paris, Paris, France

**Keywords:** Causal inference, Time-dependent confounding, Longitudinal data, Marginal structural models, Multitherapy

## Abstract

**Background:**

The Marginal Structural Cox Model (Cox-MSM), an alternative approach to handle time-dependent confounder, was introduced for survival analysis and applied to estimate the joint causal effect of two time-dependent nonrandomized treatments on survival among HIV-positive subjects. Nevertheless, Cox-MSM performance in the case of multiple treatments has not been fully explored under different degree of time-dependent confounding for treatments or in case of interaction between treatments. We aimed to evaluate and compare the performance of the marginal structural Cox model (Cox-MSM) to the standard Cox model in estimating the treatment effect in the case of multiple treatments under different scenarios of time-dependent confounding and when an interaction between treatment effects is present.

**Methods:**

We specified a Cox-MSM with two treatments including an interaction term for situations where an adverse event might be caused by two treatments taken simultaneously but not by each treatment taken alone. We simulated longitudinal data with two treatments and a time-dependent confounder affected by one or the two treatments. To fit the Cox-MSM, we used the inverse probability weighting method. We illustrated the method to evaluate the specific effect of protease inhibitors combined (or not) to other antiretroviral medications on the anal cancer risk in HIV-infected individuals, with CD4 cell count as time-dependent confounder.

**Results:**

Overall, Cox-MSM performed better than the standard Cox model. Furthermore, we showed that estimates were unbiased when an interaction term was included in the model.

**Conclusion:**

Cox-MSM may be used for accurately estimating causal individual and joined treatment effects from a combination therapy in presence of time-dependent confounding provided that an interaction term is estimated.

**Electronic supplementary material:**

The online version of this article (10.1186/s12874-017-0434-1) contains supplementary material, which is available to authorized users.

## Background

Combining multiple treatments is a common practice in the therapeutic strategy of chronic or infectious diseases in order to strengthen the effect of treatments or to limit the resistance of pathogens to therapies. When an adverse event occurs in a patient taking multiple treatments, the mainstay of therapy would be to discontinue the suspected inducing drug while maintaining others. In this case, precise identification of the causative treatment is essential.

This topic is particularly relevant when performing a safety analysis of treatments in cohort studies. In such studies, the presence of time-dependent confounders (i.e. covariates that predict disease progression and treatment initiation) affected by past treatment might lead to biased estimates if conventional regression methods are used [1, 2]. Furthermore, estimating the individual effect of each treatment becomes methodologically challenging when given simultaneously and when treatment changes overtime.

Marginal structural models (MSMs), a class of causal models, have been proposed as a solution to estimate the causal effect of a time-dependent treatment in the presence of time-dependent confounders [3, 4]. In this approach, the inverse probability of treatment weighted (IPTW) estimation method is used to consistently estimate MSM parameters [5]. In the context of multiple treatments, a seminal work introduced Cox-MSM for survival analysis and applied it to estimate the joint causal effect (efficacy) of two time-dependent nonrandomized treatments on survival among HIV-positive subjects [6]. IPTW estimation was used to compute stabilized weights related to multiple medication intakes and to balance the treatment groups at each month. The statistically significant beneficial effects observed were consistent with the results of previous randomized clinical trials. More recently, IPTW estimation was used for joint treatment effects of two treatments or the marginal effect of one treatment in a setting where two concurrent treatments are given at a point in time [7]. MSMs have been used to study the direct effect of several exposures on the outcome of interest by controlling interrelation over time between studied exposures and between exposures and time-dependent covariates. In this way, Tager et al. [8] simultaneously studied the effects of physical activity and body composition on functional limitation in the elderly by controlling the confusion induced by interrelation of these two variables over time and their relation to other covariates. To more clearly illustrate the differences in methods and their influence on bias, they carried out simulations comparing weighted and unweighted analysis with respect to the true parameters. However, they did not consider interaction in their simulation study. Howe et al. used joint Cox-MSM to estimate the joint effects of multiple time-varying exposures (alcohol consumption and injected drug use) on HIV acquisition [9]. Lopez-Gatell et al. used a joint Cox-MSM to estimate the effect of incident tuberculosis disease and Highly Active Antiretroviral therapy (HAART) initiation on AIDS-related mortality [10]. Cole et al. estimated the joint effects of HAART and PCP prophylaxis on time to AIDS or death using marginal structural models [11]. Bodnar et al. estimated the causal effect of 16 different combinations (regimes) of iron treatment throughout pregnancy on the odds of anemia at delivery [12].

Nevertheless, to date, Cox-MSM performance in the case of multiple treatments has not been fully explored under different degree of time-dependent confounding for treatments or in case of interaction between treatments. While other studies [8, 9, 11] have included interaction between treatments in their analyses, none has specifically focused on the bias generated when interaction is excluded from the estimated model. This latter issue is critical as numerous adverse events are caused by specific drug-drug interactions and would not occur if each drug was taken separately (e.g., interactions with cytochrome P450 3A4 inhibitors and statins).

The goal of this paper is to evaluate the Cox-MSM performance for estimating the individual and joined effects of multiple treatments when they are given in combination through simulation studies. For the sake of simplicity we will limit our study to exploring the use of two treatments with a potential interaction between treatments. We will compare results from Cox-MSM with estimates obtained using a classic time-dependent Cox regression model and provide an application in the context of HIV infection to evaluate the specific effect of protease inhibitors combined (or not) to other antiretroviral medications on the risk of anal cancer in HIV-infected individuals, using CD4 cell count as time-dependent confounder.

The paper is structured as follows: Section 2 describes the method used in this work; Section 3 provides the results of simulation studies that estimate the individual effects of two treatments on an adverse event; Section 4 presents an application of the method. We discuss our results in section 5 and finally, we conclude in section 6.

## Methods

### Notation for the cox-MSM

We considered a longitudinal study in which n subjects (labeled *i* = 1, …, n) entered a study at baseline, given multiple treatments and were followed at regular time intervals from enrollment into the cohort up to M visits or until the event of interest. Visits (labeled m = 0, 1, 2,…, M) were assumed to take place at the beginning of intervals in the form [m, m + 1]. At each interval, the value of the time-dependent confounder, the treatments and the event were observed. We used capital letters to represent random variables and lower-case letters to represent possible realizations (values) of random variables. We explored the model with one disease progression marker and considered the case where a subject might be given two treatments. A_1i_ (m) and A_2i_ (m) denote dichotomous variables indicating whether patient I received treatment A_1_ and/or A_2_ at visit m. Accordingly, there are four possible categories for treatment exposure: exposed to both treatments (A_1i_ (m), A_2i_ (m)) = (1, 1), exposed to only one treatment (A_1i_ (m), A_2i_ (m)) = (1, 0) or (A_1i_ (m), A_2i_ (m)) = (0, 1), not exposed (A_1i_ (m), A_2i_ (m)) = (0, 0). We denoted the baseline fixed covariates V = L (0), the time-dependent confounder by L_i_ (m), the event (death or side effect) by Y_i_ (m) and the associated failure time variable that may either be exactly observed or interval censored by T_i_. We used overbar to represent a covariate history up to a visit, i.e. ${\overline{A}}_i$ (m) = (A_i_ (0), A_i_ (1), … A_i_ (m)) and ${\overline{L}}_i$ (m) = (L_i_ (0), L_i_ (1), … L_i_ (m)) to indicate treatment and confounder history up to visit m.

### The cox-MSM with two treatments

We specified the Cox-MSM when two treatments are given to a patient:

1 $$ {\lambda}_{T_{\overline{a}}}\left(m|V\right)={\lambda}_0(m)\ \exp \left({\beta}_1{a}_1(m)+{\beta}_2{a}_2(m)+{\beta}_3{a}_1(m){a}_2(m)+{\beta}_4V\right) $$

Where T is the random variable representing a subject’s survival time given the treatment history, ${\lambda}_{T_{\overline{a}}}\left(m|V\right)$ is the hazard of T at visit m among subjects given pretreatment covariates V, *λ*_0_(*m*) is the unspecified baseline hazard at visit m, exp.(*β*_1_), exp.(*β*_2_) and exp.(*β*_3_) are the causal rate ratios for each treatment and their interaction, and exp.(*β*_4_) is the rate ratio associated with the vector of baseline covariates.

### Simulation method

We performed simulations using a cohort of HIV-positive individuals receiving multiple antiretroviral treatments. We set CD4 count as the time-dependent confounding covariate L_i_ and occurrence of an adverse event as outcome Y_i_. We simulated a data structure where the outcome at visit m depended on current treatment status only. We assumed that the only baseline covariate was the pre-treatment value of the confounder L_i_ (0). This section presents data generation and structure.

**Fig. 1:**
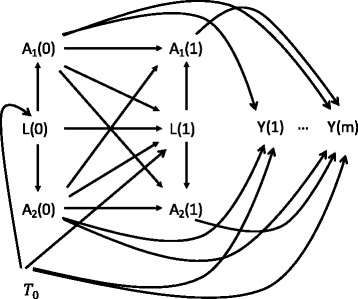
Causal directed acyclic graphs corresponding to the structure of simulated data. A_1_ and A_2_ are the treatments, L is the time-dependent confounder and Y is the outcome. Case 1 and 2 considered all relationship between A_1_, A_2_ and L. The time-dependent Confounder was strongly associated to the treatments A_1_ and A_2_ in the case 1 whereas it was weakly associated to the treatments A_2_ in the case 2. Coefficients of the time-dependent confounder in the functions of treatment prediction were set to 0.004 and 0.001, respectively. Case 3: relationship between A_2_ and L were not considered. Data were simulated from a marginal structural model as the confounding in the exposures-outcome relationship arises via T_0_ as follows: Y (m + 1) ← T_0_ → L (m) → A_1_ (m), Y (m + 1) ← T_0_ → L (m) → A_2_ (m)

We assumed that: A_1i_ (m), A_2i_ (m) and L_i_ (m) remained constant during the subsequent interval between visits m and (m + 1); treatment continued once initiated; and there was no loss to follow-up - thus we considered the case where censoring occurred only at the end of the follow-up. Figure 1 shows the causal directed acyclic graph corresponding to the structure of simulated data. We considered three different cases of time-dependent confounding. In case 1 and 2, the two treatments were predicted by the time-dependent confounder and affected the future value of the time-dependent confounder but the coefficient of the time-dependent confounder in the function of treatment prediction was set to different values for treatment A_2_ in case 1 (= strong confounding) and case 2 (= weak confounding). In case 3, treatment A_2_ was not predicted by the time-dependent confounder, which was not affected by that treatment.

Several studies simulated data from Cox-MSM under different conditions [13–20]. In our study, we simulated data for two treatments, adapting the data generation processes for one treatment described in Young et al. and Vourli and Touloumi [15, 21]. For each simulated subject we generated: (a) counterfactual survival times, T_i_^0^ from an exponential distribution with parameter λ; (b) covariate values at baseline (time 0) as follows: L_i_ (0) = b + c log T_i_^0^ + e_i,0_, where b ∼ N(μ _b_, σ_b_^2^); e_i_,_m_ ∼ N(0, σ_e_^2^); c is the coefficient that gives the strength of association between the confounder time-dependent covariate and the counterfactual survival time; (c) treatments A_1i_ (m) and A_2i_ (m) from a distribution conditional on a function of past variables. For each treatment this function included L_i_ (m), A_1i_ (m-1), A_2i_ (m-1) and the product of A_1i_ (m-1) and A_2i_ (m-1). For each m, we generated subsequent values of the covariate L_i_ (m) as a linear function of past variables; (d) finally, we generated the actual survival time T_i_ of each individual.

We considered five different sets of parameters (A, B, C, D, E) for the marginal true effects of treatments on the outcome resulting in a total of 15 sub-cases (numbered 1A to 3E). Furthermore, for the cases 1 and 2, coefficient of the time-dependent confounder in the function of treatment prediction (see Additional file 1) were set to 0.004 (α_1_ = 0.004, ω_1_ = 0.004) and 0.001 (α_1_ = 0.004, ω_1_ = 0.001), implying a strong and a weak confounding, respectively (Table 1). The coefficient c that gives the strength of association between the time-dependent confounder and the counterfactual survival time was set to 6 and μ _b_ and σ_b_ were equal to 600 and 200, respectively. σ_c_ was equal to 3.

**Table 1:** Parameters of marginal true effect for the 15 simulated sub-cases

Situations	Case 1 α_1_ = 0.004, ω_1_ = 0.004 Strong confounding for A_2_	Case 2 α_1_ = 0.004, ω_1_ = 0.001 Weak confounding for A_2_	Case 3 α_1_ = 0.004, ω_1_ = 0.000 No confounding for A_2_
A	β_1_ = 0.5, β_2_ = 0.5, β_3_ = 0	β_1_ = 0.5, β_2_ = 0.5, β_3_ = 0	β_1_ = 0.5, β_2_ = 0.5, β_3_ = 0
B	β_1_ = 0.5, β_2_ = 0.5, β_3_ = 0.5	β_1_ = 0.5, β_2_ = 0.5, β_3_ = 0.5	β_1_ = 0.5, β_2_ = 0.5, β_3_ = 0.5
C	β1 = 0, β2 = 0.5, β3 = 0	β1 = 0, β2 = 0.5, β3 = 0	β1 = 0, β2 = 0.5, β3 = 0
D	β_1_ = 0, β_2_ = 0, β_3_ = 0.5	β_1_ = 0, β_2_ = 0, β_3_ = 0.5	β_1_ = 0, β_2_ = 0, β_3_ = 0.5
E	β_1_ = 0, β_2_ = 0, β_3_ = 0	β_1_ = 0, β_2_ = 0, β_3_ = 0	β_1_ = 0, β_2_ = 0, β_3_ = 0

α_1_ and ω_1_ are the coefficients of the time-dependent confounder in the functions of prediction for treatment A_1_ and A_2_, respectively

The event rate varied between 0.1% to 2%. To avoid separation of data, simulations with no event in at least one category of treatment exposure were discarded.

For each set of parameters, we generated 1000 datasets with 1000 patients each. We assumed that each patient in this cohort was followed for 12 months and had monthly clinical follow-up visits.

### Estimation of the cox MSM with two treatments

To fit the Cox-MSM and in order to keep the weight variability as low as possible, the stabilized weights (SW) for estimation via IPTW were used in all analyses [4]. Weights related to each treatment and final censoring were computed and multiplied to obtain a final set of weights as follows:

2 $$ {\boldsymbol{SW}}_{\boldsymbol{i}}^{{\boldsymbol{A}}_1}\left(\boldsymbol{m}\right)=\prod \limits_{\boldsymbol{m}=1}^{\boldsymbol{M}}\frac{\boldsymbol{P}\left[{\boldsymbol{A}}_{1\boldsymbol{i}}\left(\boldsymbol{m}\right)|{\overline{\boldsymbol{A}}}_{1\boldsymbol{i}}\left(\boldsymbol{m}-1\right),{\overline{\boldsymbol{A}}}_{2\boldsymbol{i}}\left(\boldsymbol{m}-1\right),{\boldsymbol{L}}_{\boldsymbol{i}}(0)\right]}{\boldsymbol{P}\left[{\boldsymbol{A}}_{1\boldsymbol{i}}\left(\boldsymbol{m}\right)|{\overline{\boldsymbol{A}}}_{1\boldsymbol{i}}\left(\boldsymbol{m}-1\right),{\overline{\boldsymbol{A}}}_{2\boldsymbol{i}}\left(\boldsymbol{m}-1\right),{\overline{\boldsymbol{L}}}_{\boldsymbol{i}}\left(\boldsymbol{m}\right)\right]} $$

3 $$ {\boldsymbol{SW}}_{\boldsymbol{i}}^{{\boldsymbol{A}}_2}\left(\boldsymbol{m}\right)=\prod \limits_{\boldsymbol{m}=1}^{\boldsymbol{M}}\frac{\boldsymbol{P}\left[{\boldsymbol{A}}_{2\boldsymbol{i}}\left(\boldsymbol{m}\right)|{\overline{\boldsymbol{A}}}_{1\boldsymbol{i}}\left(\boldsymbol{m}-1\right),{\overline{\ \boldsymbol{A}}}_{2\boldsymbol{i}}\left(\boldsymbol{m}-1\right),{\boldsymbol{L}}_{\boldsymbol{i}}(0)\right]}{\boldsymbol{P}\left[{\boldsymbol{A}}_{2\boldsymbol{i}}\left(\boldsymbol{m}\right)|{\overline{\boldsymbol{A}}}_{1\boldsymbol{i}}\left(\boldsymbol{m}-1\right),{\overline{\boldsymbol{A}}}_{2\boldsymbol{i}}\left(\boldsymbol{m}-1\right),{\overline{\boldsymbol{L}}}_{\boldsymbol{i}}\left(\boldsymbol{m}\right)\right]} $$

4 $$ {\boldsymbol{SW}}_{\boldsymbol{i}}^{\boldsymbol{C}}\left(\boldsymbol{m}\right)=\prod \limits_{\boldsymbol{m}=1}^{\boldsymbol{M}}\frac{\boldsymbol{P}\left[\boldsymbol{C}\left(\boldsymbol{m}\right)|{\overline{\boldsymbol{C}}}_{\boldsymbol{i}}\left(\boldsymbol{m}-1\right),{\overline{\boldsymbol{A}}}_{1\boldsymbol{i}}\left(\boldsymbol{m}-1\right),{\overline{\boldsymbol{A}}}_{2\boldsymbol{i}}\left(\boldsymbol{m}-1\right),{\boldsymbol{L}}_{\boldsymbol{i}}(0)\right]}{\boldsymbol{P}\left[\boldsymbol{C}\left(\boldsymbol{m}\right)|{\overline{\boldsymbol{C}}}_{\boldsymbol{i}}\left(\boldsymbol{m}-1\right),{\overline{\boldsymbol{A}}}_{1\boldsymbol{i}}\left(\boldsymbol{m}-1\right),{\overline{\boldsymbol{A}}}_{2\boldsymbol{i}}\left(\boldsymbol{m}-1\right),{\overline{\boldsymbol{L}}}_{\boldsymbol{i}}\left(\boldsymbol{m}\right)\right]} $$

5 $$ {\boldsymbol{SW}}_{\boldsymbol{i}}\left(\boldsymbol{m}\right)={\boldsymbol{SW}}_{\boldsymbol{i}}^{{\boldsymbol{A}}_1}\left(\boldsymbol{m}\right)\times {\boldsymbol{SW}}_{\boldsymbol{i}}^{{\boldsymbol{A}}_2}\left(\boldsymbol{m}\right)\times {\boldsymbol{SW}}_{\boldsymbol{i}}^{\boldsymbol{C}}\left(\boldsymbol{m}\right) $$

The numerator of the SW is the probability that a subject received observed treatment A_i_ at visit m conditional only on A_1i_, A_2i_ history and baseline covariate. The denominator is the probability that a subject received observed treatment A_i_ at visit m given A_1i_, A_2i_ history and time-dependent covariate.

Once the weights were computed, we fitted a weighted Cox proportional hazard model to estimate parameters [9]. We used robust variance estimators to estimate standard errors [22]. We implemented this analysis by using the covs option in the time-dependent Phreg procedure in SAS [9]. For the situation where interaction was not set to zero, we also examined the model without including the interaction term. To assess the performance of the model, we computed the absolute bias defined as the difference between average simulated estimates and its corresponding true values and the coverage rate defined as the percentage of confidence intervals that included the true value.

## Results

**Fig. 2:**
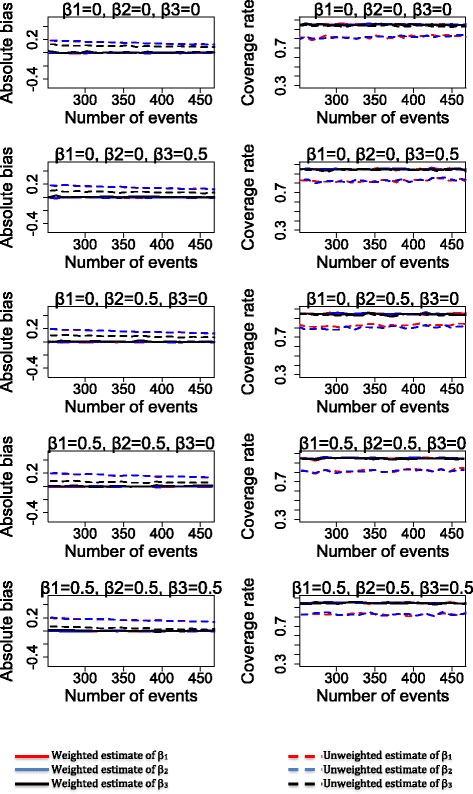
Bias and coverage rate of treatment effects estimates for the sub-cases 1A, 1B, 1C, 1D and 1E

**Fig. 3:**
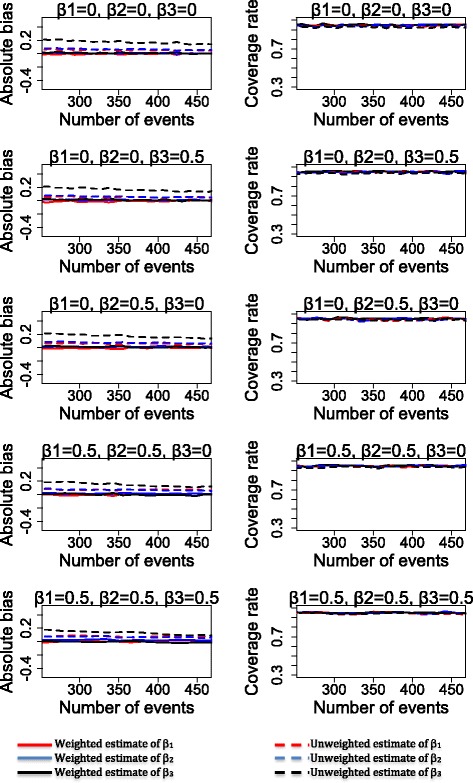
Bias and coverage rate of treatment effects estimates for the sub-cases 2A, 2B, 2C, 2D and 2E

**Fig. 4:**
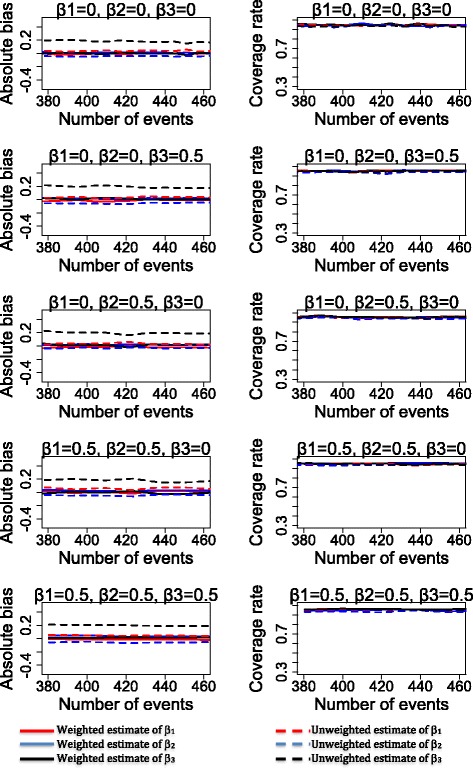
Bias and coverage rate of treatment effects estimates for the sub-cases 3A, 3B, 3C, 3D and 3E

Figures 2, 3 and 4 show the bias and the 95% coverage rate of unweighted and weighted treatment effect estimates as the number of events increases for the different cases. Values of mean bias (MB), standard deviations of estimates, root mean squared error (RMSE) and mean coverage rate (MCR) for all cases are presented in the supplementary material.

As shown in Figs. 2, 3 and 4 (see Additional file 2: Table S1 for mean values), weighted analysis yielded the most accurate estimates of the treatment effects. Indeed, weighted analysis yielded unbiased estimates for the treatment effects A_1_, A_2_ and interaction between treatments in all cases. In contrast, estimates of unweighted analysis were clearly biased in case 1 (for treatment effect A_1_ and A_2_), case 2 and case 3 (for interaction between treatments). Estimates of unweighted analysis were less biased for interaction between treatment (in case1) and treatment effects A_1_, A_2_ (in case 2 and case 3). The values of standard deviations were different from RMSE in all cases of unweighted analysis while the weighted analysis produced values of standard deviation identical to that of the RMSE. Furthermore, for estimates of the unweighted analysis, we observed a slight decrease of bias value as the number of events increased in case1 (for treatment effects A_1_ and A_2_ and interaction between treatments), case 2 and 3 (only for interaction between treatments).

**Fig. 5:**
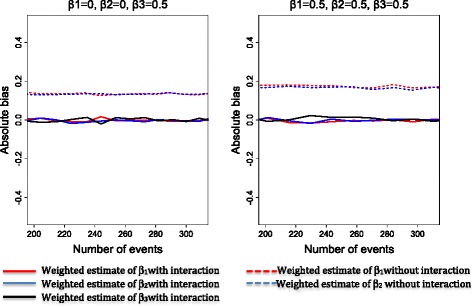
Bias of treatment effects estimates for the sub-cases 1B and 1D according to whether interaction was estimated in the model

For sub-cases 1B and 1E, weighted estimates obtained when interaction was not included in the model were biased compared to those obtained when the interaction was included in the model (Fig. 5).

## Application to exploring the risk of anal cancer associated with exposure to protease inhibitor in HIV-1 infected persons from the FHDH-ANRS CO4 cohort

Recently, Bruyand et al. [23] found a possible association of cumulative PI (protease inhibitor) exposure with a higher risk of anal cancer in HIV-1 infected persons. However, these primary analyses did not adjust for CD4 count at treatment initiation and duration of CD4 count <200 cells/μl, known to be associated with the likelihood of receiving PI and the risk of anal cancer [24]. We applied the Cox-MSM framework to evaluate the individual and joined effects of PIs given in combination with other antiretroviral treatments (ARVs), on the risk of anal cancer in HIV1-infected persons. Data were obtained from the FHDH cohort (French Hospital Database on HIV-ANRS CO4), a nationwide hospital cohort initiated in 1989 for individuals infected with HIV [25]. We selected all HIV 1-infected treatment naïve individuals at enrollment until 2008. Demographic, clinical, laboratory, ARV information, and cancer events were collected at enrollment and at follow-up visits as reported elsewhere [26]. For illustration purposes, all ARVs other than PIs were grouped in a single category irrespective of drug class. Baseline covariates were age, gender, transmission group, origin (sub-Saharan vs other), AIDS diagnosis at baseline, CD4 cell count and HIV RNA. Time-dependent covariates were AIDS diagnosis, CD4 cell count, HIV RNA. The time-dependent confounder was the CD4 cell count. The follow-up was split into one-month periods. Treatment and all time-dependent covariates were assumed to remain constant within each period. Time zero was the enrollment date in FHDH. Patients were followed until the occurrence of anal cancer, death or the end of follow-up, whichever occurred first. A total of 72,355 patients (531,823 person-years) were followed. The median age of the study population was 34 years at enrollment in FHDH. Study subjects were 67% male and 79% from Sub-Saharan origin. Median CD4 cell count and HIV RNA at baseline were 360 cells/μL and 10,095 copies/mL, respectively. The cohort experienced 9972 person-years (PY) of PIs only, 237,323 PY of other ARV and 130,428 PY of PIs and other ARVs, given simultaneously. During the follow-up, a total of 130 patients (24/100,000 PY) developed anal cancer. The rate of anal cancer was 90/100,000 PY for patients who received PIs only, 27/100,000 PY for those who received other ARVs, 33/100,000 PY for those who received PIs and other ARVs and 9.6/100,000 PY for untreated patients.

To determine whether current CD4 count predicted treatment with PIs and other ARVs, we fitted pooled logistic models for treatment initiation with PIs and other ARVs that included the baseline covariates and the time dependent covariates. CD4 cell count predicted treatment with PIs (Odds-ratios (OR) = 2.77 (*p* < .0001) for low (<200 cells/μL) versus high CD4 cell count (> 500 cells/μL) and OR = 1.39 (p < .0001) for moderate (200–500 cells/μL) versus high CD4 cell count). CD4 cell count also predicted treatment with other ARVs (OR = 5.63 (p < .0001) for low versus high, and 2.00 (p < .0001) for moderate versus high CD4 cell count, respectively). To determine whether the treatments had an impact on the CD4 count, we fitted a linear model for the mean CD4 count (in cells/μl) in the current month given the baseline covariates, PIs and other ARVs in the previous month, and the remaining time-dependent covariates in the previous month [6]. As expected, we found that PIs and other ARVs have an impact on the CD4 count, with coefficient estimates by the linear model of 1.44 (p < .0001) and 0.89 (p < .0001), respectively. This exploratory analysis confirmed that CD4 was a potential time-dependent confounder affected by past treatment exposure as described in case 1.

**Fig. 6:**
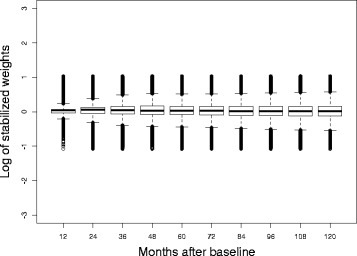
Distribution of stabilized weights related to PIs and other ARVs

Stabilized weights, related to each treatment class (PIs, other ARVs) and censoring, were then constructed using Eqs. (2), (3) and (4). They were estimated using logistic regression models with baseline covariates for the SW numerator and baseline and time-dependent covariates for the SW denominator. To reduce the impact of extremely high weights, we truncated the weights at the 1st and the 99th percentiles of their distribution across all person-months of follow-up [27]. The SW had a mean of 1.10 and a standard error of 0.37 after truncation at the 1st and the 99th percentiles (Fig. 6).

For the Cox-MSM, in addition to treatment variables including interaction, we adjusted for baseline covariates – this weighted model should be considered as the reference model. We also fitted a weighted model without the interaction term. For the standard time dependent Cox model, we adjusted for all baseline covariates and time-dependent covariates and interaction was estimated. The product term between PIs and other ARVs would represent interaction in these models only in the absence of bias due to confounding or selection. Conversely, the product term would represent effect measure modification if bias due to confounding was present for only 1 of the 2 treatments [9, 28].

Table 2 presents estimates of hazard ratios for treatment variables. In the reference model, the risk of anal cancer was significantly increased in patients with isolated PI therapy. Based on the weighted model without interaction, none of PIs, other ARVs nor the combination increased the risk of anal cancer. Conversely, all treatment variables appeared to be associated with the risk of anal cancer in the time-dependent Cox model – leading to potentially spurious conclusions. Other variables associated with increased risk of anal cancer in the reference model were longer cumulative duration with CD4 count <200 cells/μl and being a MSM (Men who have sex with Men vs Women) – (results not shown – see Additional file 3: Table S2). Our findings suggest that an increased risk of anal cancer, if any, may exist in the specific category of patients taking PI monotherapy.

**Table 2:** Comparison of estimates of HR for ARV obtained by Cox-MSM and standard time-dependent Cox models

	^a^Weighted model with interaction			Weighted model without interaction			Time dependent Cox model with interaction		
Covariates	Hazard ratio	95% CI	*P* value	Hazard ratio	95% CI	*P* value	Hazard ratio	95% CI	*P* value
PI alone vs no treatment	3.99	1.55–10.3	**0.004**	1.15	0.76–1.74	0.52	3.79	1.53–9.43	**0.004**
Other ARV alone vs no treatment	1.77	0.91–3.42	0.09	1.15	0.68–1.97	0.60	1.92	1.02–3.61	**0.04**
PI and Other ARV vs no treatment	1.69	0.84–3.39	0.14	1.32	0.76–2.31	0.33	1.90	1.00–3.68	**0.05**

Hazard ratios for the causal effects of ARV combinations with and w/o PI versus no treatment on the risk of anal cancer in HIV-infected persons followed for 6,381,871 person-months

^a^Reference method

Bold data indicate that the test was statistically significant

## Discussion

Through simulation study, we explored the performance of the Cox MSM for estimating the individual effects of two treatments given simultaneously. The simulations showed that using a joint Cox-MSM in the presence of a time varying confounder yielded unbiased estimates while standard time-dependent Cox model yielded biased estimates. Furthermore, we showed the importance of estimating the interaction term when exploring treatment effects from combination therapy.

The strength of our simulation study is twofold: first, we generated data that is suitable for analysis by a Cox-MSM and secondly, we applied a data generation process to simulate data for two treatments, while Vourli and Touloumi [15] and Young et al. [15, 21] performed simulations for only one treatment. Furthermore, we generated a data structure where both combined treatments depend on each other by including an interaction term between both treatments in the treatment predictive model. We also considered a realistic situation when a specific adverse event might be caused by two treatments taken simultaneously but not by one treatment taken alone.

Our simulation study has several limitations. First, we considered that the hazard depends only on the current treatments status. However, treatment effects may cumulate over time and depend on the time since exposure [29]. This requires an assessment as to whether the treatment effects cumulate over time when estimating the individual and joined effects of treatments given in combination [18]. Furthermore, with only one time-dependent confounder, our simulated setting could be considered unrealistic and too simplistic. Further studies are needed to consider more complex simulated settings with multiple time-dependent confounders and complex hazard functions (cumulative treatment). A number of studies have proposed various algorithms of simulating data suitable for fitting Cox-MSMs [14, 17, 30] and could be useful in this context. Second, we explored situations where only two treatments or two classes of treatment were administered; however in real life a patient could receive more co-medications. Applying this framework to a real situation with more than two treatments could make calculations of stabilized weights more complex as one has to consider multiple and complex interactions between all treatments. Third, our simulations suggested that our results and conclusions are robust with respect to the number of simulated events, and treatment or confounder effects on the hazards. Future simulations should investigate wider ranges of these parameters as well as the potential impact of the sample size, impact of missing values or unmeasured confounder on the results. Fourth, our result confirmed the superiority of the Cox-MSM on the standard time-dependent Cox model. Other methods could be explored to estimate the individual effect of treatments when given in combination. For example: doubly robust estimation [31, 32], combines inverse probability weighting with regression modeling of the relationship between covariates and the outcome for each treatment in such a way that, as long as either the propensity score or the regression models are correctly specified, the effect of exposure on the outcome will be correctly estimated. Other methods (e.g., targeted maximum likelihood estimation [33, 34], g-computation, g-estimation of structural nested models etc.. [35]) are also potential alternatives. In addition, other choices for estimating weights in multiple treatment settings could be used, e.g. multinomial logistic regression or machine learning methods [36]. Taken together, future studies would be needed to compare our results with these alternative methods. Fifth, comparing the Cox-MSM parameter estimate to any conditional treatment effect estimate is not straightforward when non-collapsible measures, such as hazard ratio or odds ratio, are employed [17, 37–39]. We did not perform numerical experiments to explore how the marginal and conditional estimates could differ, which is a limitation of our study. However, the difference between the conditional and marginal parameters is expected to be negligible as the event rate in the time intervals under consideration was small [17, 38].

Exchangeability, positivity and correct model specification are three conditions for unbiased estimation of Cox-MSM [6, 9, 27, 28]. For the exchangeability, we assumed that the selected covariates are sufficient to adjust for both confounding and selection bias. The limitation is that this is not testable in an observational study. In our study, we did not observe departures from positivity assumption after truncation of weights as the latter is based on a lack of extreme weights [27]. The lack of extreme weights obtained after truncation provides some evidence against model misspecification.

Using the weighted model with interaction, we found a significant association between use of PIs alone and the risk of anal cancer in HIV infected persons. The HR estimates were markedly different from those obtained with the weighted model without interaction – a finding due to a significant interaction between PI and other ARVs (β3 = −1.43 in the weighted model). Compared with the time dependent Cox model, HR estimate for PIs alone was higher in the weighted model with interaction while HR for other treatment variables were lower leading to different conclusions based on statistical testing – however HR from these two models were in the same range of values. This indicates that time-dependent confounding might be weak for all treatment variables and that the time dependent Cox dependent estimates are only slightly biased. In previous studies, Bruyand et al. and Chao et al. found an association between PIs use and anal cancer risk [23, 40]. In both cases, multivariable Poisson models were used. The first study did not adjust for CD4 count at initiation and/or cumulative duration of CD4 count <200 cells/μl and the second one adjusted for CD4 count as time-dependent covariate but none dealt with complex time-dependent confounding. In our application, we used the model (weighted model with interaction) that performed more accurately in our simulations. Nevertheless, the limitation of our application is that we did not take into account the cumulative duration of ARV exposure. This requires further analysis with cumulative duration of ARV as exposure.

In summary, we evaluated the joint Cox-MSM for estimating the individual and joined effects of treatments given in combination in observational studies. The Cox-MSM performed accurately in a simulation study under all scenarios. Furthermore, the Cox-MSM did not perform accurately when an interaction term was not considered in the model. The application of the framework (weighted model with interaction) on real longitudinal data confirmed the results obtained in the simulation study and has shown the utility of the Joint Cox-MSM for estimating the individual and joined causal effects of treatments when they are given in combination in observational studies.

## Conclusion

Cox-MSM may be used for accurately estimating causal individual and joined treatment effects from a combination therapy in presence of time-dependent confounding provided that an interaction term is estimated.

## Additional files

Additional file 1: Complete data generation. (DOCX 28 kb)

Additional file 2: Table S1. Mean bias, standard deviation, mean squared error and mean coverage rate of estimates. (DOCX 75 kb)

Additional file 3: Tables S2. Multivariate parameter estimates for covariate association with the risk of anal cancer in HIV-infected persons: comparison of weighted Cox MSM and standard time dependent Cox models. (DOCX 22 kb)

## Abbreviations

AIDS: Acquired immune deficiency syndrome
ANRS: Agence Nationale de Recherche sur le Sida et les hépatites virales
ARVs: Antiretroviral treatments
COX-MSM: Marginal structural Cox model
FHDH: French Hospital Database on HIV
HIV: Human immunodeficiency virus infection
IPTW: Inverse Probability of treatment weighted
MB: Mean bias
MCR: Mean coverage rate
MSM: Men who have sex with men
MSMs: Marginal structural models
PI: Protease inhibitor
PY: Person-years
RMSE: Root mean squared error
RNA: Ribonucleic acid
SW: Stabilized weights
