# Supplementary material for: Performance of the marginal structural cox model for estimating individual and joined effects of treatments given in combination
Source: BMC Med Res Methodol. 2017 Dec 4;17:160. doi: 10.1186/s12874-017-0434-1 (PMC5715511; doi:10.1186/s12874-017-0434-1)
Supplement: Supplementary file 1 — Complete data generation. (DOCX 28 kb) [file 12874_2017_434_MOESM1_ESM.docx]

# Complete data generation

For each i = 1, …, n, we defined A_i_ (-1) = Y_i_ (-1) = 0 and we did the following:

1. We generated counterfactual survival times, T^0^ from an exponential distribution with parameter λ.
2. We generated covariate values at time 0 as follow:

L_i_ (0) = b + c log T_i_^0^ + e_i,0_,

where b ~ N(μ, σ_e_^2^), e_i_,_m_ ~ N(0, σ_e_^2^).

For each time point m,

1. Treatments A_1i_ and A_2i_ at visit m, were simulated as a function of the treatments A_1i_, A_2i_ at the previous visit m-1, interaction between both treatments at the previous visit m-1, current covariate value L_i_ at the visit m and baseline covariate :

**Logit** (**P [A_1i_ (m) |** ${\bar{\boldsymbol{A}}}_{\boldsymbol{1}\boldsymbol{i}}\left( \boldsymbol{m-1} \right)$**,** ${\bar{\boldsymbol{A}}}_{\boldsymbol{2}\boldsymbol{i}}\left( \boldsymbol{m-1} \right)$**,** ${\bar{\boldsymbol{L}}}_{\boldsymbol{i}}\left( \boldsymbol{m-1} \right)$**, V, Y (m) = 0]) = α_0_ +** **α_1_ L_i_ (m) + α_2_ A_1i_ (m-1) + α_3_ A_2i_ (m-1) + α_4_ A_1i_ (m-1) A_2i_ (m-1) + α_5_ V**

**Logit (P [A_2i_ (m) |** ${\bar{\boldsymbol{A}}}_{\boldsymbol{1}\boldsymbol{i}}\left( \boldsymbol{m-1} \right)$**,** ${\bar{\boldsymbol{A}}}_{\boldsymbol{2}\boldsymbol{i}}\left( \boldsymbol{m-1} \right)$**,** ${\bar{\boldsymbol{L}}}_{\boldsymbol{i}}\left( \boldsymbol{m-1} \right)$**, V, Y (m) = 0]) = ω_0_ +** **ω_1_ L_i_ (m) + ω_2_ A_1i_ (m-1) + ω_3_ A_2i_ (m-1) + ω_4_ A_1i_ (m-1) A_2i_ (m-1) + ω_5_ V**

1. Subsequent values of the covariate L_i_ (m) at visit m were generated as a function of the value of covariate L_i_ (m-1) and both the treatments A_1i_ (m-1) and A_2i_ (m-1) at previous visit as follow:

L_i_ (m) = L_i_ (m-1) + θ_1_ A_1i_ (m-1) + θ_2_ A_2i_ (m-1) + c log T_i_^0^ + (e_i_,_m_)

1. Finally, we generated the failure status Y_i_ (m+1) and the actual survival time T_i_ of each individual as follows:

The survival time T_i_ under a given regime a (m) [a_1_ (m), a_2_ (m)] is computed from the cumulative hazard $\int_{0}^{m+1} \lambda_{\mathrm{ai}}\left( i \right)\mathrm{di}$

λa (m)= exp [β_1_a_1i_ (m) + β2 a_2i_(m) + β_3_ a_1i_ (m) a_2i_ (m)]

At each step of the data generation, this cumulative hazard is updated based on the new am value, accumulating the risk for the regime A (m)=a (m). As the counterfactual survival times T_i0_ and T_i_ follow the same distribution if either β_1_=0, β_2_=0, β_3_=0 or A_1_=0, A_2_=0, T_i0_ is compared with the calculated T_i_ to determine whether the subject fails in the next interval, i.e., if T_i0_ is greater than the calculated T_i_ then the failure indicator Y_i_ (m+1)=0; otherwise Y_i_ (m+1)=1.
