# Supplementary material for: Performance of the marginal structural cox model for estimating individual and joined effects of treatments given in combination
Source: BMC Med Res Methodol. 2017 Dec 4;17:160. doi: 10.1186/s12874-017-0434-1 (PMC5715511; doi:10.1186/s12874-017-0434-1)
Supplement: Supplementary file 2 — Mean bias, standard deviation, mean squared error and mean coverage rate of estimates. (DOCX 75 kb) [file 12874_2017_434_MOESM2_ESM.docx]

# Table S1. Mean bias, standard deviation, mean squared error and mean coverage rate of estimates

| Case1  Number of events = 260-300 | | | | | | | | | | |
| --- | --- | --- | --- | --- | --- | --- | --- | --- | --- | --- |
|  |  | **Bias A_1_**  **SD**  **(Min-max)** | **Bias A_2_**  **SD**  **(Min-max)** | **Bias A_1_A_2_**  **SD**  **(Min-max)** | **RMSE A_1_** | **RMSE A_2_** | **RMSE A_1_A_2_** | **CR A_1_** | **CR A_2_** | **CR A_1_A_2_** |
| β_1_=0,β_2_=0,β_3_=0 | **Weighted model** | -0.01  0.19  -0.64 – 0.59 | 0.00  0.19  -0.64 – 0.62 | 0.00  0.29  -1.07 – 1.12 | 0.19 | 0.19 | 0.29 | 0.95 | 0.95 | 0.95 |
|  | **Unweighted model** | 0.17  0.17  -0.37 – 0.71 | 0.18  0.17  -0.38 – 0.74 | 0.11  0.25  -0.76 – 0.97 | 0.24 | 0.24 | 0.27 | 0.81 | 0.80 | 0.94 |
| β_1_=0,β_2_=0,β_3_=0.5 | **Weighted model** | 0.00  0.19  -0.64 – 0.67 | 0.00  0.19  -0.67 – 0.59 | 0.01  0.29  -0.88 – 0.96 | 0.19 | 0.19 | 0.29 | 0.95 | 0.95 | 0.94 |
|  | **Unweighted model** | 0.18  0.17  -0.43 – 0.74 | 0.17  0.17  -0.42 – 0.75 | 0.09  0.25  -0.71 – 0.93 | 0.25 | 0.25 | 0.27 | 0.82 | 0.82 | 0.94 |
| β_1_=0,β_2_=0.5,β_3_=0 | **Weighted model** | -0.01  0.20  -0.69 – 0.66 | 0.00  0.18  -0.66 – 0.58 | 0.01  0.28  -0.84 – 1.07 | 0.20 | 0.18 | 0.28 | 0.94 | 0.95 | 0.95 |
|  | **Unweighted model** | 0.18  0.18  -0.43 – 0.75 | 0.18  0.16  -0.36 – 0.75 | 0.10  0.25  -0.64 – 0.87 | 0.26 | 0.25 | 0.27 | 0.81 | 0.79 | 0.94 |
| β_1_=0.5,β_2_=0.5,β_3_=0 | **Weighted model** | -0.01  0.20  -0.66 – 0.63 | 0.00  0.20  -0.65 – 0.59 | 0.01  0.28  -0.85 – 0.92 | 0.20 | 0.20 | 0.28 | 0.94 | 0.95 | 0.95 |
|  | **Unweighted model** | 0.18  0.18  -0.46 – 0.75 | 0.19  0.18  -0.33 – 0.75 | 0.08  0.24  -0.67 – 0.89 | 0.26 | 0.26 | 0.25 | 0.81 | 0.80 | 0.94 |
| β_1_=0.5,β_2_=0.5,β_3_=0.5 | **Weighted model** | 0.00  0.21  -0.66 – 0.67 | -0.01  0.21  -0.70 – 0.67 | 0.01  0.28  -0.84 – 1.00 | 0.21 | 0.21 | 0.28 | 0.95 | 0.95 | 0.94 |
|  | **Unweighted model** | 0.19  0.18  -0.49 – 0.78 | 0.18  0.18  -0.34 – 0.70 | 0.06  0.24  -0.73 – 0.85 | 0.26 | 0.25 | 0.25 | 0.83 | 0.85 | 0.95 |

| Case1  Number of events = 300-340 | | | | | | | | | | |
| --- | --- | --- | --- | --- | --- | --- | --- | --- | --- | --- |
|  |  | **Bias A_1_**  **SD**  **(Min-max)** | **Bias A_2_**  **SD**  **(Min-max)** | **Bias A_1_A_2_**  **SD**  **(Min-max)** | **RMSE A_1_** | **RMSE A_2_** | **RMSE A_1_A_2_** | **CR A_1_** | **CR A_2_** | **CR A_1_A_2_** |
| β_1_=0,β_2_=0,β_3_=0 | **Weighted model** | 0.00  0.17  -0.56 – 0.60 | 0.00  0.17  -0.58 – 0.55 | 0.01  0.28  -0.84 – 0.97 | 0.17 | 0.17 | 0.28 | 0.95 | 0.95 | 0.94 |
|  | **Unweighted model** | 0.16  0.16  -0.35 – 0.70 | 0.16  0.16  -0.35 – 0.67 | 0.11  0.24  -0.68 – 0.87 | 0.23 | 0.23 | 0.26 | 0.81 | 0.81 | 0.93 |
| β_1_=0,β_2_=0,β_3_=0.5 | **Weighted model** | 0.00  0.18  -0.57 – 0.56 | 0.00  0.18  -0.59 – 0.54 | 0.01  0.26  -0.86 – 1.02 | 0.18 | 0.18 | 0.26 | 0.95 | 0.95 | 0.95 |
|  | **Unweighted model** | 0.16  0.17  -0.38 – 0.67 | 0.16  0.16  -0.38 – 0.67 | 0.08  0.23  -0.62 – 0.83 | 0.23 | 0.23 | 0.25 | 0.82 | 0.82 | 0.94 |
| β_1_=0,β_2_=0.5,β_3_=0 | **Weighted model** | -0.01  0.19  -0.70 – 0.61 | 0.00  0.17  -0.59 -0.63 | 0.01  0.27  -0.80 – 0.94 | 0.19 | 0.17 | 0.27 | 0.94 | 0.95 | 0.94 |
|  | **Unweighted model** | 0.17  0.17  -0.41 – 0.77 | 0.17  0.15  -0.38 – 0.71 | 0.09  0.24  -0.65 – 0.83 | 0.24 | 0.23 | 0.25 | 0.82 | 0.80 | 0.93 |
| β_1_=0.5,β_2_=0.5,β_3_=0 | **Weighted model** | 0.00  0.18  -0.54 – 0.62 | 0.00  0.18  -0.64 – 0.61 | 0.00  0.26  -0.91 – 0.83 | 0.18 | 0.18 | 0.26 | 0.95 | 0.95 | 0.95 |
|  | **Unweighted model** | 0.18  0.16  -0.31 – 0.74 | 0.18  0.16  -0.40 – 0.71 | 0.06  0.22  -0.70 – 0.81 | 0.24 | 0.24 | 0.23 | 0.81 | 0.81 | 0.95 |
| β_1_=0.5,β_2_=0.5,β_3_=0.5 | **Weighted model** | -0.01  0.19  -0.69 – 0.63 | 0.00  0.20  -0.62 – 0.62 | 0.00  0.26  -0.83 – 0.91 | 0.19 | 0.20 | 0.26 | 0.95 | 0.95 | 0.95 |
|  | **Unweighted model** | 0.18  0.17  -0.45 – 0.72 | 0.18  0.17  -0.39 – 0.72 | 0.05  0.23  -0.75 – 0.77 | 0.25 | 0.25 | 0.23 | 0.83 | 0.82 | 0.95 |

| Case1  Number of events = 340-380 | | | | | | | | | | |
| --- | --- | --- | --- | --- | --- | --- | --- | --- | --- | --- |
|  |  | **Bias A_1_**  **SD**  **(Min-max)** | **Bias A_2_**  **SD**  **(Min-max)** | **Bias A_1_A_2_**  **SD**  **(Min-max)** | **RMSE A_1_** | **RMSE A_2_** | **RMSE A_1_A_2_** | **CR A_1_** | **CR A_2_** | **CR A_1_A_2_** |
| β_1_=0,β_2_=0,β_3_=0 | **Weighted model** | 0.00  0.16  -0.53 – 0.54 | 0.00  0.16  -0.57 – 0.56 | 0.00  0.26  -0.81 – 0.90 | 0.16 | 0.16 | 0.26 | 0.95 | 0.95 | 0.95 |
|  | **Unweighted model** | 0.15  0.15  -0.32 – 0.65 | 0.15  0.14  -0.32 – 0.62 | 0.10  0.22  -0.66 – 0.85 | 0.21 | 0.21 | 0.24 | 0.81 | 0.82 | 0.94 |
| β_1_=0,β_2_=0,β_3_=0.5 | **Weighted model** | 0.00  0.17  -0.64 – 0.53 | 0.00  0.17  -0.56 – 0.56 | 0.01  0.25  -0.85 – 0.97 | 0.17 | 0.17 | 0.25 | 0.95 | 0.95 | 0.94 |
|  | **Unweighted model** | 0.15  0.15  -0.42 – 0.64 | 0.15  0.15  -0.37 – 0.64 | 0.08  0.22  -0.62 – 0.86 | 0.21 | 0.21 | 0.23 | 0.82 | 0.82 | 0.95 |
| β_1_=0,β_2_=0.5,β_3_=0 | **Weighted model** | -0.01  0.18  -0.64 – 0.57 | 0.00  0.16  -0.47 – 0.52 | 0.01  0.26  -0.78 – 1.00 | 0.18 | 0.16 | 0.26 | 0.95 | 0.95 | 0.94 |
|  | **Unweighted model** | 0.15  0.16  -0.40 – 0.67 | 0.16  0.14  -0.29 – 0.63 | 0.09  0.22  -0.63 – 0.85 | 0.22 | 0.21 | 0.24 | 0.83 | 0.81 | 0.94 |
| β_1_=0.5,β_2_=0.5,β_3_=0 | **Weighted model** | 0.00  0.18  -0.58 – 0.55 | 0.00  0.17  -0.58 – 0.53 | 0.00  0.25  -0.77 – 1.01 | 0.18 | 0.17 | 0.25 | 0.95 | 0.95 | 0.95 |
|  | **Unweighted model** | 0.17  0.0.16  -0.36 – 0.64 | 0.17  0.15  -0.36 – 0.65 | 0.06  0.22  -0.66 – 0.78 | 0.23 | 0.23 | 0.23 | 0.81 | 0.81 | 0.94 |
| β_1_=0.5,β_2_=0.5,β_3_=0.5 | **Weighted model** | 0.00  0.18  -0.71 – 0.61 | -0.01  0.18  -0.66 – 0.56 | 0.00  0.25  -0.77- 0.98 | 0.18 | 0.18 | 0.25 | 0.95 | 0.95 | 0.95 |
|  | **Unweighted model** | 0.16  0.17  -0.38 – 0.74 | 0.16  0.16  -0.42 – 0.67 | 0.04  0.22  -0.65 – 0.96 | 0.23 | 0.23 | 0.23 | 0.83 | 0.83 | 0.95 |

| Case1  Number of events = 380-420 | | | | | | | | | | |
| --- | --- | --- | --- | --- | --- | --- | --- | --- | --- | --- |
|  |  | **Bias A_1_**  **SD**  **(Min-max)** | **Bias A_2_**  **SD**  **(Min-max)** | **Bias A_1_A_2_**  **SD**  **(Min-max)** | **RMSE A_1_** | **RMSE A_2_** | **RMSE A_1_A_2_** | **CR A_1_** | **CR A_2_** | **CR A_1_A_2_** |
| β_1_=0,β_2_=0,β_3_=0 | **Weighted model** | 0.00  0.15  -0.58 – 0.51 | -0.01  0.15  -0.53 – 0.48 | 0.01  0.25  -0.81 – 0.84 | 0.15 | 0.15 | 0.25 | 0.95 | 0.95 | 0.95 |
|  | **Unweighted model** | 0.14  0.14  -0.33 – 0.59 | 0.14  0.14  -0.33 – 0.57 | 0.10  0.21  -0.61 – 0.80 | 0.20 | 0.20 | 0.23 | 0.82 | 0.83 | 0.94 |
| β_1_=0,β_2_=0,β_3_=0.5 | **Weighted model** | 0.00  0.16  -0.53 – 0.49 | -0.01  0.16  -0.51 – 0.50 | 0.01  0.24  -0.78 – 0.81 | 0.16 | 0.16 | 0.24 | 0.95 | 0.95 | 0.94 |
|  | **Unweighted model** | 0.14  0.14  -0.32 – 0.56 | 0.14  0.15  -0.34 – 0.59 | 0.07  0.21  -0.61 – 0.74 | 0.20 | 0.20 | 0.22 | 0.83 | 0.83 | 0.95 |
| β_1_=0,β_2_=0.5,β_3_=0 | **Weighted model** | 0.00  0.17  -0.56 – 0.59 | 0.00  0.15  -0.52 – 0.49 | 0.00  0.24  -0.73 – 0.82 | 0.17 | 0.15 | 0.24 | 0.94 | 0.95 | 0.95 |
|  | **Unweighted model** | 0.15  0.15  -0.33 – 0.62 | 0.15  0.13  -0.31 – 0.56 | 0.08  0.21  -0.60 – 0.76 | 0.21 | 0.20 | 0.22 | 0.83 | 0.81 | 0.94 |
| β_1_=0.5,β_2_=0.5,β_3_=0 | **Weighted model** | -0.01  0.16  -0.57 – 0.50 | 0.00  0.16  -0.57 – 0.61 | 0.01  0.24  -0.79 – 0.82 | 0.16 | 0.16 | 0.24 | 0.95 | 0.95 | 0.95 |
|  | **Unweighted model** | 0.15  0.14  -0.35 – 0.61 | 0.15  0.14  -0.33 – 0.64 | 0.06  0.20  -0.64 – 0.69 | 0.21 | 0.21 | 0.21 | 0.83 | 0.83 | 0.95 |
| β_1_=0.5,β_2_=0.5,β_3_=0.5 | **Weighted model** | 0.00  0.17  -0.57 – 0.54 | 0.00  0.17  -0.53 – 0.54 | 0.00  0.24  -0.71 – 0.78 | 0.17 | 0.17 | 0.24 | 0.95 | 0.95 | 0.94 |
|  | **Unweighted model** | 0.16  0.15  -0.34 – 0.70 | 0.15  0.15  -0.31 – 0.71 | 0.03  0.21  -0.62 – 0.70 | 0.22 | 0.22 | 0.21 | 0.82 | 0.83 | 0.95 |

| Case1  Number of events = 420-460 | | | | | | | | | | |
| --- | --- | --- | --- | --- | --- | --- | --- | --- | --- | --- |
|  |  | **Bias A_1_**  **SD**  **(Min-max)** | **Bias A_2_**  **SD**  **(Min-max)** | **Bias A_1_A_2_**  **SD**  **(Min-max)** | **RMSE A_1_** | **RMSE A_2_** | **RMSE A_1_A_2_** | **CR A_1_** | **CR A_2_** | **CR A_1_A_2_** |
| β_1_=0,β_2_=0,β_3_=0 | **Weighted model** | -0.01  0.15  -0.50 – 0.48 | 0.00  0.14  -0.48 – 0.47 | 0.01  0.24  -0.71 – 0.84 | 0.15 | 0.14 | 0.24 | 0.95 | 0.95 | 0.95 |
|  | **Unweighted model** | 0.13  0.13  -0.33 – 0.56 | 0.13  0.13  -0.33 – 0.51 | 0.09  0.20  -0.57 – 0.74 | 0.18 | 0.19 | 0.22 | 0.83 | 0.83 | 0.93 |
| β_1_=0,β_2_=0,β_3_=0.5 | **Weighted model** | 0.00  0.15  -0.48 – 0.48 | -0.01  0.15  -0.54 – 0.46 | 0.00  0.23  -0.72 – 0.74 | 0.15 | 0.15 | 0.23 | 0.95 | 0.95 | 0.95 |
|  | **Unweighted model** | 0.13  0.13  -0.32 – 0.59 | 0.13  0.13  -0.35 – 0.54 | 0.07  0.20  -0.54 – 0.67 | 0.19 | 0.19 | 0.19 | 0.84 | 0.85 | 0.95 |
| β_1_=0,β_2_=0.5,β_3_=0 | **Weighted model** | 0.00  0.16  -0.51 – 0.51 | 0.00  0.15  -0.46 – 0.45 | 0.00  0.23  -0.75 – 0.76 | 0.16 | 0.15 | 0.23 | 0.95 | 0.95 | 0.94 |
|  | **Unweighted model** | 0.14  0.15  -0.35 – 0.59 | 0.14  0.13  -0.28 – 0.56 | 0.07  0.20  -0.61 – 0.71 | 0.20 | 0.19 | 0.21 | 0.83 | 0.81 | 0.94 |
| β_1_=0.5,β_2_=0.5,β_3_=0 | **Weighted model** | 0.00  0.16  -0.55 – 0.47 | 0.00  0.15  -0.54 – 0.53 | 0.01  0.23  -0.71 – 0.79 | 0.16 | 0.15 | 0.23 | 0.95 | 0.95 | 0.94 |
|  | **Unweighted model** | 0.14  0.14  -0.35 – 0.59 | 0.14  0.14  -0.31 – 0.61 | 0.06  0.19  -0.59 – 0.68 | 0.20 | 0.20 | 0.20 | 0.82 | 0.82 | 0.95 |
| β_1_=0.5,β_2_=0.5,β_3_=0.5 | **Weighted model** | 0.00  0.17  -0.51 – 0.54 | 0.00  0.16  -0.54 – 0.55 | 0.00  0.23  -0.68 – 0.85 | 0.17 | 0.16 | 0.23 | 0.95 | 0.95 | 0.94 |
|  | **Unweighted model** | 0.15  0.15  -0.34 – 0.62 | 0.15  0.14  -0.35 – 0.65 | 0.03  0.20  -0.64 – 0.69 | 0.21 | 0.21 | 0.20 | 0.82 | 0.83 | 0.94 |

| Case1  Number of events = 460-500 | | | | | | | | | | |
| --- | --- | --- | --- | --- | --- | --- | --- | --- | --- | --- |
|  |  | **Bias A_1_**  **SD**  **(Min-max)** | **Bias A_2_**  **SD**  **(Min-max)** | **Bias A_1_A_2_**  **SD**  **(Min-max)** | **RMSE A_1_** | **RMSE A_2_** | **RMSE A_1_A_2_** | **CR A_1_** | **CR A_2_** | **CR A_1_A_2_** |
| β_1_=0,β_2_=0,β_3_=0 | **Weighted model** | 0.00  0.14  -0.49 – 0.45 | 0.00  0.14  -0.48 – 0.49 | 0.01  0.23  -0.73 – 0.77 | 0.14 | 0.14 | 0.23 | 0.94 | 0.95 | 0.94 |
|  | **Unweighted model** | 0.12  0.13  -0.32 – 0.53 | 0.12  0.13  -0.28 – 0.52 | 0.09  0.20  -0.57 – 0.72 | 0.18 | 0.18 | 0.22 | 0.84 | 0.83 | 0.92 |
| β_1_=0,β_2_=0,β_3_=0.5 | **Weighted model** | -0.01  0.15  -0.52 – 0.42 | 0.00  0.15  -0.47 – 0.48 | 0.00  0.22  -0.69 – 0.79 | 0.15 | 0.15 | 0.22 | 0.94 | 0.95 | 0.95 |
|  | **Unweighted model** | 0.12  0.13  -0.33 – 0.54 | 0.12  0.13  -0.32 – 0.56 | 0.07  0.19  -0.50 – 0.72 | 0.18 | 0.18 | 0.20 | 0.84 | 0.84 | 0.94 |
| β_1_=0,β_2_=0.5,β_3_=0 | **Weighted model** | 0.00  0.15  -0.51 – 0.48 | 0.00  0.14  -0.47 – 0.42 | 0.01  0.23  -0.68 – 0.76 | 0.15 | 0.14 | 0.23 | 0.95 | 0.95 | 0.94 |
|  | **Unweighted model** | 0.13  0.14  -0.33 – 0.56 | 0.12  0.12  -0.27 – 0.51 | 0.07  0.19  -0.56 – 0.65 | 0.19 | 0.18 | 0.21 | 0.84 | 0.82 | 0.93 |
| β_1_=0.5,β_2_=0.5,β_3_=0 | **Weighted model** | 0.00  0.15  -0.50 – 0.52 | -0.01  0.15  -0.44 – 0.47 | 0.01  0.22  -0.64 – 0.78 | 0.15 | 0.15 | 0.22 | 0.95 | 0.94 | 0.95 |
|  | **Unweighted model** | 0.14  0.13  -0.33 – 0.59 | 0.14  0.14  -0.31 – 0.58 | 0.06  0.19  -0.62 – 0.62 | 0.19 | 0.19 | 0.20 | 0.84 | 0.83 | 0.95 |
| β_1_=0.5,β_2_=0.5,β_3_=0.5 | **Weighted model** | -0.01  0.15  -0.51 – 0.49 | -0.01  0.15  -0.50 – 0.44 | 0.01  0.22  -0.67 – 0.79 | 0.15 | 0.15 | 0.22 | 0.95 | 0.95 | 0.94 |
|  | **Unweighted model** | 0.13  0.14  -0.32- 0.59 | 0.13  0.13  -0.29 – 0.55 | 0.03  0.19  -0.54 – 0.62 | 0.20 | 0.19 | 0.19 | 0.83 | 0.84 | 0.95 |

| Case2  Number of events = 260-300 | | | | | | | | | | |
| --- | --- | --- | --- | --- | --- | --- | --- | --- | --- | --- |
|  |  | **Bias A_1_**  **SD**  **(Min-max)** | **Bias A_2_**  **SD**  **(Min-max)** | **Bias A_1_A_2_**  **SD**  **(Min-max)** | **RMSE A_1_** | **RMSE A_2_** | **RMSE A_1_A_2_** | **CR A_1_** | **CR A_2_** | **CR A_1_A_2_** |
| β_1_=0,β_2_=0,β_3_=0 | **Weighted model** | -0.01  0.32  -1.49 – 0.92 | 0.01  0.21  -0.64 – 0.71 | 0.02  0.35  -1.02 – 1.48 | 0.33 | 0.21 | 0.35 | 0.95 | 0.94 | 0.95 |
|  | **Unweighted model** | 0.06  0.31  -1.44 – 0.93 | 0.08  0.20  -0.53 – 0.74 | 0.20  0.35  -0.77 – 1.74 | 0.32 | 0.21 | 0.40 | 0.94 | 0.93 | 0.93 |
| β_1_=0,β_2_=0,β_3_=0.5 | **Weighted model** | -0.01  0.34  -1.34 – 1.01 | 0.01  0.22  -0.65 – 0.74 | 0.01  0.36  -1.09 – 1.48 | 0.34 | 0.22 | 0.36 | 0.95 | 0.95 | 0.95 |
|  | **Unweighted model** | 0.06  0.33  -1.24 – 1.00 | 0.07  0.21  -0.56 – 0.77 | 0.19  0.36  -0.91 – 1.61 | 0.34 | 0.22 | 0.41 | 0.94 | 0.94 | 0.94 |
| β_1_=0,β_2_=0.5,β_3_=0 | **Weighted model** | -0.01  0.40  -1.84 – 1.10 | 0.02  0.23  -0.71 – 0.81 | 0.02  0.42  -1.26 – 1.92 | 0.40 | 0.23 | 0.42 | 0.95 | 0.95 | 0.95 |
|  | **Unweighted model** | 0.06  0.38  -1.77 – 1.14 | 0.09  0.22  -0.59 – 0.82 | 0.20  0.42  -1.05 – 2.11 | 0.39 | 0.23 | 0.47 | 0.94 | 0.95 | 0.94 |
| β_1_=0.5,β_2_=0.5,β_3_=0 | **Weighted model** | -0.01  0.37  -1.84 – 1.11 | 0.02  0.25  -0.72 – 0.96 | 0.01  0.39  -1.25 – 1.92 | 0.37 | 0.25 | 0.39 | 0.95 | 0.95 | 0.95 |
|  | **Unweighted model** | 0.07  0.36  -1.59 – 1.16 | 0.08  0.24  -0.62 – 0.97 | 0.19  0.39  -1.02 – 1.89 | 0.37 | 0.25 | 0.43 | 0.95 | 0.95 | 0.93 |
| β_1_=0.5,β_2_=0.5,β_3_=0.5 | **Weighted model** | 0.00  0.40  1.49 – 1.13 | 0.02  0.27  -0.75 – 0.95 | 0.00  0.41  -1.23 – 1.50 | 0.40 | 0.27 | 0.41 | 0.95 | 0.95 | 0.95 |
|  | **Unweighted model** | 0.09  0.38  -1.56 – 1.24 | 0.07  0.26  -0.67 – 1.01 | 0.16  0.41  -0.98 – 1.75 | 0.39 | 0.27 | 0.44 | 0.95 | 0.95 | 0.95 |

| Case2  Number of events = 300-340 | | | | | | | | | | |
| --- | --- | --- | --- | --- | --- | --- | --- | --- | --- | --- |
|  |  | **Bias A_1_**  **SD**  **(Min-max)** | **Bias A_2_**  **SD**  **(Min-max)** | **Bias A_1_A_2_**  **SD**  **(Min-max)** | **RMSE A_1_** | **RMSE A_2_** | **RMSE A_1_A_2_** | **CR A_1_** | **CR A_2_** | **CR A_1_A_2_** |
| β_1_=0,β_2_=0,β_3_=0 | **Weighted model** | -0.01  0.30  -1.32 – 0.93 | 0.01  0.19  -0.57 – 0.60 | 0.01  0.32  -0.95 – 1.41 | 0.30 | 0.19 | 0.32 | 0.95 | 0.94 | 0.95 |
|  | **Unweighted model** | 0.05  0.29  -1.23 – 0.93 | 0.07  0.18  -0.50 – 0.63 | 0.18  0.32  -0.76 – 1.55 | 0.29 | 0.19 | 0.37 | 0.94 | 0.94 | 0.93 |
| β_1_=0,β_2_=0,β_3_=0.5 | **Weighted model** | -0.02  0.32  -1.24 – 0.90 | 0.01  0.20  -0.64 – 0.66 | 0.02  0.34  -1.00 – 1.21 | 0.32 | 0.20 | 0.34 | 0.95 | 0.95 | 0.95 |
|  | **Unweighted model** | 0.05  0.31  -1.00 – 0.96 | 0.06  0.19  -0.54 – 0.73 | 0.18  0.34  -0.83 – 1.32 | 0.32 | 0.20 | 0.39 | 0.94 | 0.94 | 0.93 |
| β_1_=0,β_2_=0.5,β_3_=0 | **Weighted model** | -0.01  0.36  -1.31 – 1.07 | 0.01  0.21  -0.67 – 0.77 | 0.01  0.37  -1.14 – 1.34 | 0.36 | 0.21 | 0.37 | 0.95 | 0.95 | 0.95 |
|  | **Unweighted model** | 0.06  0.34  -1.20 – 1.15 | 0.07  0.20  -0.58 – 0.80 | 0.18  0.37  -1.05 – 1.53 | 0.35 | 0.22 | 0.41 | 0.95 | 0.94 | 0.95 |
| β_1_=0.5,β_2_=0.5,β_3_=0 | **Weighted model** | 0.00  0.34  -1.49 – 1.03 | 0.01  0.23  -0.63 – 0.86 | 0.00  0.35  -1.09 – 1.48 | 0.34 | 0.23 | 0.35 | 0.95 | 0.95 | 0.95 |
|  | **Unweighted model** | 0.08  0.32  -1.29 – 1.11 | 0.07  0.22  -0.55 – 0.90 | 0.16  0.35  -0.94 – 1.59 | 0.33 | 0.23 | 0.38 | 0.94 | 0.95 | 0.94 |
| β_1_=0.5,β_2_=0.5,β_3_=0.5 | **Weighted model** | 0.00  0.39  -1.71 – 1.25 | 0.03  0.25  -0.92 – 0.95 | -0.01  0.40  -1.23 – 1.72 | 0.39 | 0.26 | 0.40 | 0.94 | 0.95 | 0.95 |
|  | **Unweighted model** | 0.08  0.37  -1.65 – 1.27 | 0.08  0.24  -0.59 – 0.98 | 0.14  0.40  -1.11 – 1.96 | 0.38 | 0.25 | 0.42 | 0.93 | 0.95 | 0.94 |

| Case2  Number of events = 340-380 | | | | | | | | | | |
| --- | --- | --- | --- | --- | --- | --- | --- | --- | --- | --- |
|  |  | **Bias A_1_**  **SD**  **(Min-max)** | **Bias A_2_**  **SD**  **(Min-max)** | **Bias A_1_A_2_**  **SD**  **(Min-max)** | **RMSE A_1_** | **RMSE A_2_** | **RMSE A_1_A_2_** | **CR A_1_** | **CR A_2_** | **CR A_1_A_2_** |
| β_1_=0,β_2_=0,β_3_=0 | **Weighted model** | -0.01  0.28  -1.11 – 0.80 | 0.01  0.18  -0.55 – 0.66 | 0.01  0.30  -0.92 – 1.18 | 0.28 | 0.18 | 0.30 | 0.95 | 0.95 | 0.95 |
|  | **Unweighted model** | 0.05  0.27  -1.03 – 0.85 | 0.07  0.17  -0.45 – 0.70 | 0.17  0.30  -0.79 – 1.39 | 0.28 | 0.18 | 0.35 | 0.94 | 0.94 | 0.93 |
| β_1_=0,β_2_=0,β_3_=0.5 | **Weighted model** | -0.01  0.31  -1.29 – 0.93 | 0.01  0.19  -0.55 – 0.64 | 0.01  0.32  -1.05 – 1.44 | 0.31 | 0.19 | 0.32 | 0.95 | 0.95 | 0.95 |
|  | **Unweighted model** | 0.06  0.29  -1.20 – 0.94 | 0.06  0.18  -0.50 – 0.68 | 0.16  0.32  -0.94 – 1.53 | 0.30 | 0.19 | 0.36 | 0.94 | 0.95 | 0.94 |
| β_1_=0,β_2_=0.5,β_3_=0 | **Weighted model** | -0.01  0.35  -1.47 – 1.06 | 0.01  0.20  -0.62 – 0.64 | 0.01  0.36  -1.09 – 1.48 | 0.35 | 0.20 | 0.36 | 0.95 | 0.95 | 0.95 |
|  | **Unweighted model** | 0.06  0.34  -1.35 – 1.10 | 0.07  0.19  -0.51 - 67 | 0.17  0.36  -0.90 – 1.61 | 0.34 | 0.21 | 0.40 | 0.94 | 0.94 | 0.94 |
| β_1_=0.5,β_2_=0.5,β_3_=0 | **Weighted model** | 0.00  0.32  -1.21 – 1.05 | 0.02  0.22  -0.64 – 0.91 | 0.00  0.34  -1.14 – 1.27 | 0.32 | 0.22 | 0.34 | 0.95 | 0.95 | 0.95 |
|  | **Unweighted model** | 0.07  0.31  -1.16 – 1.07 | 0.07  0.21  -0.53 – 0.93 | 0.15  0.33  -0.94 – 1.14 | 0.32 | 0.22 | 0.37 | 0.94 | 0.94 | 0.94 |
| β_1_=0.5,β_2_=0.5,β_3_=0.5 | **Weighted model** | -0.01  0.34  -1.07 – 1.18 | 0.02  0.23  -0.64 – 0.85 | 0.01  0.35  -1.13 – 1.02 | 0.34 | 0.23 | 0.35 | 0.95 | 0.95 | 0.95 |
|  | **Unweighted model** | 0.07  0.32  -1.00 – 1.24 | 0.06  0.22  -0.59 – 0.90 | 0.14  0.35  -0.92 – 1.14 | 0.33 | 0.23 | 0.37 | 0.95 | 0.95 | 0.95 |

| Case2  Number of events = 380 - 420 | | | | | | | | | | |
| --- | --- | --- | --- | --- | --- | --- | --- | --- | --- | --- |
|  |  | **Bias A_1_**  **SD**  **(Min-max)** | **Bias A_2_**  **SD**  **(Min-max)** | **Bias A_1_A_2_**  **SD**  **(Min-max)** | **RMSE A_1_** | **RMSE A_2_** | **RMSE A_1_A_2_** | **CR A_1_** | **CR A_2_** | **CR A_1_A_2_** |
| β_1_=0,β_2_=0,β_3_=0 | **Weighted model** | 0.00  0.27  -1.05 – 0.77 | 0.01  0.17  -0.51 -0.55 | 0.00  0.29  -0.83 – 1.21 | 0.27 | 0.17 | 0.29 | 0.94 | 0.95 | 0.94 |
|  | **Unweighted model** | 0.05  0.26  -0.94 – 0.78 | 0.06  0.16  -0.46 – 0.56 | 0.16  0.29  -0.67 – 1.28 | 0.26 | 0.17 | 0.33 | 0.94 | 0.94 | 0.92 |
| β_1_=0,β_2_=0,β_3_=0.5 | **Weighted model** | -0.01  0.29  -1.08 – 0.85 | 0.00  0.18  -0.60 – 0.59 | 0.01  0.30  -0.91 – 1.14 | 0.29 | 0.18 | 0.30 | 0.95 | 0.95 | 0.95 |
|  | **Unweighted model** | 0.05  0.28  -1.04 – 0.86 | 0.05  0.17  -0.52 – 0.61 | 0.15  0.30  -0.75 – 1.23 | 0.28 | 0.18 | 0.34 | 0.94 | 0.94 | 0.94 |
| β_1_=0,β_2_=0.5,β_3_=0 | **Weighted model** | 0.00  0.32  -1.41 – 0.84 | 0.02  0.18  -0.53 – 0.76 | 0.00  0.33  -0.96 – 1.53 | 0.32 | 0.19 | 0.33 | 0.95 | 0.95 | 0.95 |
|  | **Unweighted model** | 0.06  0.31  -1.36 – 0.86 | 0.07  0.18  -0.44 – 0.77 | 0.15  0.33  -0.82 – 1.60 | 0.32 | 0.19 | 0.37 | 0.94 | 0.94 | 0.94 |
| β_1_=0.5,β_2_=0.5,β_3_=0 | **Weighted model** | 0.01  0.29  -1.07 – 0.97 | 0.01  0.20  -0.63 – 0.70 | -0.01  0.31  -1.03 – 1.21 | 0.29 | 0.20 | 0.31 | 0.95 | 0.95 | 0.95 |
|  | **Unweighted model** | 0.08  0.28  -0.95 – 1.04 | 0.07  0.19  -0.53 – 0.69 | 0.13  0.30  -0.93 – 1.30 | 0.29 | 0.20 | 0.33 | 0.94 | 0.95 | 0.94 |
| β_1_=0.5,β_2_=0.5,β_3_=0.5 | **Weighted model** | 0.01  0.32  -1.17 – 0.90 | 0.02  0.22  -0.60 -0.82 | -0.01  0.33  -0.90 – 1.23 | 0.32 | 0.22 | 0.33 | 0.95 | 0.95 | 0.95 |
|  | **Unweighted model** | 0.08  0.30  -1.05 – 0.93 | 0.06  0.21  -0.52 – 0.85 | 0.12  0.32  -0.77 – 1.35 | 0.32 | 0.22 | 0.35 | 0.94 | 0.95 | 0.95 |

| Case2  Number of events = 420 - 460 | | | | | | | | | | |
| --- | --- | --- | --- | --- | --- | --- | --- | --- | --- | --- |
|  |  | **Bias A_1_**  **SD**  **(Min-max)** | **Bias A_2_**  **SD**  **(Min-max)** | **Bias A_1_A_2_**  **SD**  **(Min-max)** | **RMSE A_1_** | **RMSE A_2_** | **RMSE A_1_A_2_** | **CR A_1_** | **CR A_2_** | **CR A_1_A_2_** |
| β_1_=0,β_2_=0,β_3_=0 | **Weighted model** | 0.00  0.25  -0.86 – 0.74 | 0.00  0.16  -0.50 – 0.54 | 0.00  0.27  -0.84 – 0.91 | 0.25 | 0.16 | 0.27 | 0.95 | 0.95 | 0.95 |
|  | **Unweighted model** | 0.05  0.24  -0.79 – 0.78 | 0.05  0.15  -0.44 – 0.57 | 0.15  0.27  -0.71 – 1.08 | 0.24 | 0.16 | 0.31 | 0.94 | 0.94 | 0.93 |
| β_1_=0,β_2_=0,β_3_=0.5 | **Weighted model** | 0.00  0.26  -1.00 – 0.82 | 0.01  0.16  -0.48 – 0.51 | 0.00  0.28  -0.84 – 1.97 | 0.26 | 0.16 | 0.28 | 0.95 | 0.95 | 0.95 |
|  | **Unweighted model** | 0.05  0.25  -0.93 – 0.86 | 0.05  0.16  -0.43 – 0.54 | 0.14  0.28  -0.74 – 1.19 | 0.26 | 0.17 | 0.31 | 0.94 | 0.94 | 0.94 |
| β_1_=0,β_2_=0.5,β_3_=0 | **Weighted model** | -0.01  0.29  -1.25 – 0.83 | 0.01  0.18  -0.51 – 0.60 | 0.01  0.31  -0.97 – 1.26 | 0.29 | 0.18 | 0.31 | 0.95 | 0.95 | 0.95 |
|  | **Unweighted model** | 0.05  0.28  -1.11 – 0.84 | 0.06  0.17  -0.45 – 0.66 | 0.15  0.31  -0.85 – 1.38 | 0.28 | 0.18 | 0.34 | 0.95 | 0.94 | 0.94 |
| β_1_=0.5,β_2_=0.5,β_3_=0 | **Weighted model** | 0.01  0.28  -0.95 – 0.87 | 0.01  0.19  -0.57 – 0.64 | -0.01  0.29  -0.95 – 0.97 | 0.28 | 0.19 | 0.29 | 0.95 | 0.95 | 0.95 |
|  | **Unweighted model** | 0.07  0.27  -0.81 – 0.92 | 0.06  0.18  -0.50 – 0.68 | 0.12  0.29  -0.83 – 1.07 | 0.28 | 0.19 | 0.31 | 0.94 | 0.95 | 0.94 |
| β_1_=0.5,β_2_=0.5,β_3_=0.5 | **Weighted model** | 0.02  0.30  -1.10 – 0.95 | 0.02  0.20  -0.54 – 0.77 | -0.02  0.31  -0.94 – 1.19 | 0.30 | 0.20 | 0.31 | 0.95 | 0.95 | 0.95 |
|  | **Unweighted model** | 0.08  0.29  -0.95 – 0.99 | 0.06  0.20  -0.47 – 0.75 | 0.10  0.31  -0.83 – 1.26 | 0.30 | 0.21 | 0.32 | 0.94 | 0.95 | 0.95 |

| Case2  Number of events = 460 - 500 | | | | | | | | | | |
| --- | --- | --- | --- | --- | --- | --- | --- | --- | --- | --- |
|  |  | **Bias A_1_**  **SD**  **(Min-max)** | **Bias A_2_**  **SD**  **(Min-max)** | **Bias A_1_A_2_**  **SD**  **(Min-max)** | **RMSE A_1_** | **RMSE A_2_** | **RMSE A_1_A_2_** | **CR A_1_** | **CR A_2_** | **CR A_1_A_2_** |
| β_1_=0,β_2_=0,β_3_=0 | **Weighted model** | 0.00  0.24  -0.88 – 0.73 | 0.00  0.15  -0.50 – 0.52 | 0.00  0.26  -0.82 – 0.89 | 0.24 | 0.15 | 0.26 | 0.95 | 0.95 | 0.95 |
|  | **Unweighted model** | 0.05  0.23  1.04E-8 -0.84 | 0.05  0.15  -0.44 – 0.54 | 0.13  0.26  -0.68 – 0.99 | 0.24 | 0.15 | 0.29 | 0.94 | 0.94 | 0.93 |
| β_1_=0,β_2_=0,β_3_=0.5 | **Weighted model** | 0.00  0.25  -0.83 – 0.72 | 0.01  0.16  -0.55 – 0.53 | 0.00  0.27  -0.85 – 0.90 | 0.25 | 0.16 | 0.27 | 0.95 | 0.95 | 0.95 |
|  | **Unweighted model** | 0.05  0.24  -0.74 – 0.75 | 0.05  0.15  -0.49 – 0.55 | 0.13  0.27  -0.70 – 1.03 | 0.25 | 0.16 | 0.30 | 0.93 | 0.95 | 0.94 |
| β_1_=0,β_2_=0.5,β_3_=0 | **Weighted model** | 0.00  0.29  -1.08 – 0.85 | 0.01  0.17  -0.50 – 0.60 | 0.00  0.30  -0.96 – 1.11 | 0.28 | 0.17 | 0.30 | 0.95 | 0.95 | 0.95 |
|  | **Unweighted model** | 0.06  0.27  -0.94 – 0.85 | 0.06  0.16  -0.43 – 0.62 | 0.13  0.30  -0.81 – 1.13 | 0.28 | 0.17 | 0.32 | 0.94 | 0.94 | 0.94 |
| β_1_=0.5,β_2_=0.5,β_3_=0 | **Weighted model** | 0.00  0.26  -0.93 – 0.85 | 0.01  0.18  -0.51 – 0.71 | 0.00  0.27  -0.90 – 0.97 | 0.26 | 0.18 | 0.27 | 0.95 | 0.95 | 0.95 |
|  | **Unweighted model** | 0.06  0.25  -0.85 – 0.92 | 0.05  0.17  -0.46 – 0.73 | 0.12  0.27  -0.70 – 1.07 | 0.26 | 0.18 | 0.30 | 0.94 | 0.95 | 0.94 |
| β_1_=0.5,β_2_=0.5,β_3_=0.5 | **Weighted model** | 0.01  0.29  -0.96 – 0.89 | 0.02  0.19  -0.54 – 0.80 | -0.01  0.30  -0.98 – 1.04 | 0.29 | 0.19 | 0.30 | 0.95 | 0.95 | 0.94 |
|  | **Unweighted model** | 0.07  0.28  -0.86 – 0.96 | 0.06  0.19  -0.49 – 0.81 | 0.09  0.30  -0.91 – 1.04 | 0.29 | 0.20 | 0.31 | 0.93 | 0.94 | 0.94 |

| Case3  Number of events = 380 - 420 | | | | | | | | | | |
| --- | --- | --- | --- | --- | --- | --- | --- | --- | --- | --- |
|  |  | **Bias A_1_**  **SD**  **(Min-max)** | **Bias A_2_**  **SD**  **(Min-max)** | **Bias A_1_A_2_**  **SD**  **(Min-max)** | **RMSE A_1_** | **RMSE A_2_** | **RMSE A_1_A_2_** | **CR A_1_** | **CR A_2_** | **CR A_1_A_2_** |
| β_1_=0,β_2_=0,β_3_=0 | **Weighted model** | -0.01  0.35  1.44 – 1.05 | 0.01  0.20  -0.60 – 0.73 | 0.00  0.37  -1.09 – 1.45 | 0.35 | 0.20 | 0.37 | 0.95 | 0.95 | 0.95 |
|  | **Unweighted model** | 0.03  0.35  -1.38 – 1.10 | -0.05  0.20  -0.65 – 0.70 | 0.19  0.37  -0.91 – 1.64 | 0.35 | 0.20 | 0.42 | 0.94 | 0.94 | 0.94 |
| β_1_=0,β_2_=0,β_3_=0.5 | **Weighted model** | -0.02  0.41  -1.82 – 1.05 | 0.01  0.22  -0.65 – 0.87 | 0.01  0.42  -1.13 – 1.85 | 0.41 | 0.22 | 0.42 | 0.95 | 0.95 | 0.95 |
|  | **Unweighted model** | 0.03  0.41  -1.74 – 1.06 | -0.06  0.22  -0.71 – 0.80 | 0.21  0.42  -0.93 – 2.03 | 0.41 | 0.23 | 0.47 | 0.95 | 0.94 | 0.95 |
| β_1_=0,β_2_=0.5,β_3_=0 | **Weighted model** | -0.02  0.44  -2.05 – 1.16 | 0.02  0.23  -0.67 – 0.83 | 0.01  0.45  -1.15 – 2.09 | 0.44 | 0.23 | 0.45 | 0.95 | 0.95 | 0.95 |
|  | **Unweighted model** | 0.04  0.43  -2.02 – 1.20 | -0.03  0.23  -0.72 – 0.77 | 0.20  0.45  -0.94 – 2.32 | 0.43 | 0.23 | 0.49 | 0.95 | 0.94 | 0.95 |
| β_1_=0.5,β_2_=0.5,β_3_=0 | **Weighted model** | 0.00  0.43  -1.83 – 1.22 | 0.02  0.26  -0.83 – 0.90 | 0.00  0.44  -1.17 – 1.90 | 0.43 | 0.26 | 0.44 | 0.95 | 0.95 | 0.95 |
|  | **Unweighted model** | 0.06  0.42  -1.84 – 1.23 | -0.05  0.26  -0.92 – 0.83 | 0.19  0.44  -1.03 – 2.09 | 0.43 | 0.27 | 0.48 | 0.95 | 0.93 | 0.95 |
| β_1_=0.5,β_2_=0.5,β_3_=0.5 | **Weighted model** | -0.01  0.60  -9.40 – 1.74 | 0.04  0.31  -0.86 – 1.31 | 0.01  0.61  -1.78 – 9.41 | 0.59 | 0.31 | 0.61 | 0.95 | 0.95 | 0.95 |
|  | **Unweighted model** | 0.05  0.59  -9.40 – 1.77 | 0.06  0.31  -0.98 – 1.25 | 0.21  0.61  -1.64 – 9.66 | 0.59 | 0.32 | 0.64 | 0.95 | 0.93 | 0.95 |

| Case3  Number of events = 420 - 460 | | | | | | | | | | |
| --- | --- | --- | --- | --- | --- | --- | --- | --- | --- | --- |
|  |  | **Bias A_1_**  **SD**  **(Min-max)** | **Bias A_2_**  **SD**  **(Min-max)** | **Bias A_1_A_2_**  **SD**  **(Min-max)** | **RMSE A_1_** | **RMSE A_2_** | **RMSE A_1_A_2_** | **CR A_1_** | **CR A_2_** | **CR A_1_A_2_** |
| β_1_=0,β_2_=0,β_3_=0 | **Weighted model** | 0.00  0.33  1.20 – 0.93 | 0.01  0.18  -0.60 – 0.63 | -0.01  0.34  -0.97 – 1.26 | 0.33 | 0.19 | 0.36 | 0.95 | 0.94 | 0.95 |
|  | **Unweighted model** | 0.04  0.32  -1.15 – 0.96 | -0.04  0.19  -0.66 - 59 | 0.17  0.34  -0.79 – 1.46 | 0.32 | 0.19 | 0.38 | 0.95 | 0.94 | 0.94 |
| β_1_=0,β_2_=0,β_3_=0.5 | **Weighted model** | 0.00  0.37  -1.57 – 0.96 | 0.02  0.20  -0.67 – 0.77 | 0.00  0.39  -1.01 – 1.62 | 0.37 | 0.20 | 0.39 | 0.95 | 0.95 | 0.95 |
|  | **Unweighted model** | 0.04  0.37  -1.53 – 1.00 | -0.05  0.21  -0.76 – 0.70 | 0.18  0.39  -0.82 – 1.78 | 0.37 | 0.21 | 0.43 | 0.95 | 0.94 | 0.95 |
| β_1_=0,β_2_=0.5,β_3_=0 | **Weighted model** | -0.01  0.47  -4.53 – 1.19 | 0.02  0.22  -0.59 – 0.81 | 0.01  0.48  -1.26 – 4.59 | 0.47 | 0.22 | 0.48 | 0.95 | 0.95 | 0.95 |
|  | **Unweighted model** | 0.04  0.46  -4.49 – 1.26 | -0.03  0.22  -0.63 – 0.78 | 0.19  0.48  -1.03 – 4.81 | 0.46 | 0.22 | 0.51 | 0.95 | 0.94 | 0.95 |
| β_1_=0.5,β_2_=0.5,β_3_=0 | **Weighted model** | 0.01  0.41  -1.89 – 1.21 | 0.03  0.24  -0.64 – 1.06 | -0.01  0.42  -1.34 – 2.01 | 0.41 | 0.24 | 0.42 | 0.95 | 0.95 | 0.95 |
|  | **Unweighted model** | 0.06  0.40  -1.90 – 1.26 | -0.04  0.24  -0.73 – 1.00 | 0.17  0.42  -1.15 – 2.13 | 0.41 | 0.25 | 0.45 | 0.94 | 0.94 | 0.94 |
| β_1_=0.5,β_2_=0.5,β_3_=0.5 | **Weighted model** | -0.01  0.47  -1.94 – 1.41 | 0.03  0.28  -0.82 – 1.10 | 0.01  0.48  -1.45 – 1.94 | 0.47 | 0.28 | 0.48 | 0.95 | 0.95 | 0.95 |
|  | **Unweighted model** | 0.04  0.46  -1.81 – 1.43 | -0.06  0.28  -0.92 – 1.04 | 0.19  0.48  -1.26 – 2.09 | 0.46 | 0.29 | 0.51 | 0.95 | 0.94 | 0.95 |

| Case3  Number of events = 460 - 500 | | | | | | | | | | |
| --- | --- | --- | --- | --- | --- | --- | --- | --- | --- | --- |
|  |  | **Bias A_1_**  **SD**  **(Min-max)** | **Bias A_2_**  **SD**  **(Min-max)** | **Bias A_1_A_2_**  **SD**  **(Min-max)** | **RMSE A_1_** | **RMSE A_2_** | **RMSE A_1_A_2_** | **CR A_1_** | **CR A_2_** | **CR A_1_A_2_** |
| β_1_=0,β_2_=0,β_3_=0 | **Weighted model** | 0.00  0.32  -1.34 – 0.93 | 0.01  0.17  -0.56 – 0.63 | 0.00  0.33  -0.99 – 1.34 | 0.32 | 0.18 | 0.33 | 0.95 | 0.95 | 0.95 |
|  | **Unweighted model** | 0.04  0.31  -1.32 – 0.94 | -0.04  0.18  -0.60 – 0.58 | 0.16  0.33  -0.82 – 1.52 | 0.31 | 0.18 | 0.37 | 0.94 | 0.94 | 0.94 |
| β_1_=0,β_2_=0,β_3_=0.5 | **Weighted model** | -0.01  0.35  -1.48 – 0.97 | 0.01  0.19  -0.61 – 0.70 | 0.00  0.36  -1.06 – 1.53 | 0.35 | 0.19 | 0.36 | 0.95 | 0.95 | 0.95 |
|  | **Unweighted model** | 0.04  0.34  -1.47 – 1.03 | -0.05  0.19  -0.69 – 0.65 | 0.16  0.36  -0.84 – 1.68 | 0.34 | 0.20 | 0.40 | 0.95 | 0.94 | 0.95 |
| β_1_=0,β_2_=0.5,β_3_=0 | **Weighted model** | -0.01  0.39  -2.01 -1.10 | 0.02  0.20  -0.62 – 0.76 | 0.01  0.41  -1.11 – 2.06 | 0.39 | 0.20 | 0.41 | 0.95 | 0.95 | 0.95 |
|  | **Unweighted model** | 0.03  0.39  -1.93 – 1.11 | -0.03  0.20  -0.65 – 0.70 | 0.17  0.41  -0.95 – 2.19 | 0.39 | 0.20 | 0.44 | 0.94 | 0.95 | 0.95 |
| β_1_=0.5,β_2_=0.5,β_3_=0 | **Weighted model** | 0.01  0.38  -1.81 – 1.23 | 0.03  0.23  -0.60 – 0.83 | -0.02  0.39  -1.17 – 1.83 | 0.38 | 0.23 | 0.39 | 0.95 | 0.95 | 0.95 |
|  | **Unweighted model** | 0.06  0.38  -1.78 – 1.23 | -0.04  0.23  -0.67 – 0.79 | 0.15  0.39  -1.02 – 1.98 | 0.38 | 0.23 | 0.42 | 0.95 | 0.95 | 0.94 |
| β_1_=0.5,β_2_=0.5,β_3_=0.5 | **Weighted model** | -0.01  0.45  -2.19 – 1.39 | 0.03  0.26  -0.76 – 1.16 | 0.02  0.46  -1.50 – 2.26 | 0.45 | 0.26 | 0.46 | 0.95 | 0.95 | 0.95 |
|  | **Unweighted model** | 0.04  0.44  -2.12 – 1.45 | -0.05  0.26  -0.84 – 1.07 | 0.18  0.45  -1.30 – 2.41 | 0.45 | 0.27 | 0.49 | 0.95 | 0.94 | 0.95 |
