# Supplementary material for: Performance of the marginal structural cox model for estimating individual and joined effects of treatments given in combination
Source: BMC Med Res Methodol. 2017 Dec 4;17:160. doi: 10.1186/s12874-017-0434-1 (PMC5715511; doi:10.1186/s12874-017-0434-1)
Supplement: Supplementary file 3 — Multivariate parameter estimates for covariate association with the risk of anal cancer in HIV-infected persons: comparison of weighted Cox MSM and standard time dependent Cox models. (DOCX 22 kb) [file 12874_2017_434_MOESM3_ESM.docx]

**Table S2. Multivariate parameter estimates for covariate association with the risk of anal cancer in HIV-infected persons: comparison of weighted Cox MSM and standard time dependent Cox models**

|  | Weighted model | | | Time dependent Cox model | | |
| --- | --- | --- | --- | --- | --- | --- |
| Covariates | **Parameter Estimate** | **Standard Error** | **P Value** | **Parameter Estimate** | **Standard Error** | **P value** |
| PI (β_1_) | 1.39 | 0.48 | **0.004** | 1.33 | 0.46 | **0.004** |
| Other ARV (β_2_) | 0.57 | 0.34 | 0.09 | 0.65 | 0.32 | **0.04** |
| Interaction PI and Other ARV (β_3_) | -1.43 | 0.52 | **0.01** | -1.35 | 0.50 | **0.01** |
| Cumulative duration with CD4 count < 200 cells/μl (month) | 0.03 | 0.01 | **<.0001** | 0.001 | 0.0001 | **<0.0001** |
| Cumulative duration with HIV RNA > 5 log_10_ copies/ml (month) | -0.01 | 0.01 | 0.55 | 0.0002 | 0.0001 | 0.06 |
| Age  <40  ≥40 | 0  0.34 | 0.20 | 0.09 | 0  0.45 | 0.21 | **0.04** |
| Sex and transmission group  Women  MSM  Other men | 0  1.24  0.35 | 0.28  0.31 | **<0.0001**  0.26 | 0  1.18  0.27 | 0.27  0.29 | **<0.0001**  0.34 |
| Origin  Other  Sub-saharan | 0  -1.33 | 0.71 | 0.06 | 0  -1.22 | 0.71 | 0.09 |
| AIDS status | 0.28 | 0.31 | 0.37 | 0.57 | 0.20 | **0.004** |
